# Does the Rainbow Trout Ovarian Fluid Promote the Spermatozoon on Its Way to the Egg?

**DOI:** 10.3390/ijms22179519

**Published:** 2021-09-01

**Authors:** Vitaliy Kholodnyy, Borys Dzyuba, Marek Rodina, Hermes Bloomfield-Gadêlha, Manabu Yoshida, Jacky Cosson, Sergii Boryshpolets

**Affiliations:** 1 South Bohemian Research Center of Aquaculture and Biodiversity of Hydrocenoses, Faculty of Fisheries and Protection of Waters, University of South Bohemia in České Budějovice, 289 25 Vodňany, Czech Republic; bdzyuba@frov.jcu.cz (B.D.); rodina@frov.jcu.cz (M.R.); hermes.gadelha@bristol.ac.uk (H.B.-G.); jacosson@gmail.com (J.C.); sboryshpolets@frov.jcu.cz (S.B.); 2 Department of Engineering Mathematics and Bristol Robotics Laboratory, University of Bristol, Bristol BS8 1UB, UK; 3 Misaki Marine Biological Station, School of Science, The University of Tokyo, Misaki, Miura 238-0225, Kanagawa, Japan; yoshida@mmbs.s.u-tokyo.ac.jp

**Keywords:** sperm motility, fertilization, ovarian fluid, chemotaxis, *Oncorhynchus mykiss*

## Abstract

The fertilization of freshwater fish occurs in an environment that may negatively affect the gametes; therefore, the specific mechanisms triggering the encounters of gametes would be highly expedient. The egg and ovarian fluid are likely the major sources of these triggers, which we confirmed here for rainbow trout (*Oncorhynchus mykiss*). The ovarian fluid affected significantly spermatozoa performance: it supported high velocity for a longer period and changed the motility pattern from tumbling in water to straightforward moving in the ovarian fluid. Rainbow trout ovarian fluid induced a trapping chemotaxis-like effect on activated male gametes, and this effect depended on the properties of the activating medium. The interaction of the spermatozoa with the attracting agents was accompanied by the “turn-and-run” behavior involving asymmetric flagellar beating and Ca^2+^ concentration bursts in the bent flagellum segment, which are characteristic of the chemotactic response. Ovarian fluid created the optimal environment for rainbow trout spermatozoa performance, and the individual peculiarities of the egg (ovarian fluid)–sperm interaction reflect the specific features of the spawning process in this species.

## 1. Introduction

Sexual reproduction is the fertilization of female ova by male spermatozoa. For successful fertilization, the spermatozoa must reach the egg soon after ovulation. Externally fertilizing freshwater fish spermatozoa are activated after direct contact with freshwater, and their motility is sustained for only a very limited period, typically tens of seconds [1]. Freshwater fish eggs are large compared to mammalian oocytes and possess a specialized fertilization site, namely, a micropyle, which is to be found by the diminutive sperm cell during its short motility period in an environment that is constantly moving and changing. Under these conditions, reproductive success is limited to the ability of spermatozoa to find the egg and reach the fertilization site; thus, it depends on mechanisms that increase the probability of a sperm–egg encounter.

What are these mechanisms based on? Generally, ideas vary from the predominance of a male factor, such as the “fair raffle” concept, in which the success of a particular male depends only on the number of spermatozoa it can provide to the “fertilization lottery” [2], to a specific female post-mating control, e.g., “cryptic female choice” [3]. Finally, the combination of many factors, including environmental ones such as the guidance hypothesis, are considered [4]. Numerous assumptions claim that the gametes’ encounter is controlled by stimuli or signaling from the egg or accompanying female fluids, and confirmation has been found across many taxa, including marine invertebrates, mammals, and a few fish species [5,6]. These stimuli may affect the behavior of sperm cells, creating both chemokinetic and chemotactic effects and, finally, fertilization [7].

The first observations of sperm activation and rise in motility traits in the vicinity of the eggs were made in marine invertebrates about 100 years ago [8], and “egg jelly”, a substance surrounding the eggs, was believed to be responsible for that. Marine invertebrates remain the main subjects in studies on gamete interaction, including chemotactic issues. In particular, the most popular and complete spermatozoon chemotaxis models are based on sea urchins [9] and ascidians [10]. In fish, such a female effect provider can be the ovarian fluid, which bathes mature fish oocytes in the ovarian cavity [11] and surrounds the eggs of many externally fertilizing female fish during the release of eggs through the oviducts into the water [12]. The composition of ovarian fluid among fish species varies, consisting of ions and protein, sugars, and lipids in different ratios [13,14,15].

Several reports about the chemokinetic reactions of fish male gametes, i.e., changes in spermatozoa velocity and trajectory depending on the presence of ovarian fluid in the medium [16,17,18,19] were scrutinized recently by Myers et al. [20] in their meta-analysis. Interestingly, most of the studies were conducted on salmonids, which is not surprising considering their popularity as a research object for fish reproduction studies and that they are preconditioned by wide usage in aquaculture and have a high market value. The meta-analysis [20] showed the overall enhancing effect of ovarian fluid on the velocity of salmonid spermatozoa; however, it showed the high heterogeneity of the data. Nevertheless, there is still very little information beyond these kinetic observations. It is not clear if the ovarian fluid of salmonid fish provokes any chemotactic effect or how it triggers the success of egg fertilization. There is also no clear understanding of the mechanisms of motility enhancement provided by ovarian fluid.

In our study, we performed comprehensive testing of the interaction between spermatozoa and ovarian fluid. Using a representative of the freshwater spawning Salmonidae family, the rainbow trout (*Oncorhynchus mykiss*), we searched for any evidence of chemotactic behavior to determine the potential mechanism of the gametes’ encounter guidance.

To reach our goal, we sought to answer the following questions:

- How does the presence of ovarian fluid affect the outcome of in vitro fertilization?
- What is the effect of ovarian fluid in the activation medium on the spermatozoa motility traits, including velocity and linearity of motion?
- Does ovarian fluid have a chemotactic effect on spermatozoa?
- Are changes in spermatozoa motility associated with specific features of the ovarian fluid, including osmolarity and Ca^2+^ ions?

## 2. Results

### 2.1. Ovarian Fluid Enhances the In Vitro Fertilization Outcome

It has already been demonstrated that ovarian fluid increases the success of in vitro fertilization in salmonids such as the Caspian brown trout (*Salmo trutta caspius*) [21]. We performed our in vitro fertilization test to confirm this effect in *O. mykiss*. Moreover, we considered the potential variability of the effect depending on the relatedness of the mates, in particular, if they belonged to different populations as in the case of guppies (*Poecilia reticulata*) [22] and lake trout (*Salvelinus namaycush*) [23]. In our study, one population was represented by albino males and females originating from one long-term broodstock, and the other consisted of regularly colored males originating from one broodstock. The albino type was recessive; that is, the fertilization of an albino female egg by spermatozoon from a regularly colored male resulted in a regularly color embryo/larva, which allowed a simple assessment of the sire.

**Figure 1:**
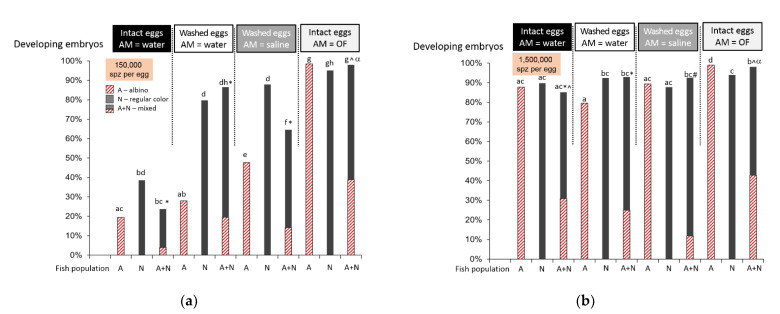
Ovarian fluid enhances in vitro fertilization. The albino rainbow trout embryo development rate after in vitro fertilization depends on the presence of ovarian fluid (OF) around the eggs or in the activation medium (AM) and male type (albino or regular color). Eggs (mixed from three females) were either non-washed to remove ovarian fluid (“intact eggs”) or washed thrice with 0.9% NaCl solution (“washed eggs”). Spermatozoa ((**a**) 0.5 µL, 150,000 per egg and (**b**) 5 µL, 1,500,000 per egg in the activation medium) from five albino males (A), five regular color males (N), or the mixed sample (A + N) were activated in 8 mL of water, isotonic NaCl saline, or ovarian fluid (AM = water, saline, or ovarian fluid, correspondingly). Then, they were added to beakers containing eggs and mixed, and the beakers were shaken for 1 min. After that, the eggs were rinsed, transferred to glass Petri dishes, and put into incubators at 11 °C. After 11 days, the developing embryos were counted, the fertilization rate was calculated (fertilized eggs/total amount of eggs), and the color type of the embryos was established. The striped bars show the albino embryos; solid bars denote regular color embryos. The statistical significance of the differences was tested using the binomial model; due to multiple comparisons, the threshold *p*-value was set to 0.0005; different superscripts denote significant differences: Latin letters, for overall development rates; the symbols * and ^ for the albino embryo share between the mixed groups; and α for the absence of a significant difference in the expected 50/50 share between albino and normal color embryos in the mixed groups.

During preliminary motility tests, it appeared that sperm from albino males had low motility in water, only up to 20%; however, the motility in the ovarian fluid was higher than 95%. Sperm from regularly colored males had more than 50% motility in water and more than 95% in the ovarian fluid. We used two concentrations of spermatozoa in the activation medium, adding 0.5 or 5 µL of sperm to 8 mL of activating medium. Fertilization of the intact (non-washed) eggs in water with a lower concentration of spermatozoa (0.5 µL, i.e., 150,000 spermatozoa per egg) resulted in a relatively low developing embryo count: less than 20% for albino males and around 40% for normal males (Figure 1a). In the case of mixed sperm, the outcome was about 20% fertilization, and only 16% of them were albino embryos. Washing eggs to remove ovarian fluid followed by fertilization in water resulted in a significant rise in fertilization with sperm from normal males (*p* < 0.0005, binomial test with *p*-value corrected for multiple comparisons) and no significant changes for the albino male group (*p* = 0.01). The amount of fertilized eggs in the mixed group also rose (*p* < 0.0005), while the share of albino embryos stayed the same (*p* = 0.28); nevertheless, their absolute number was higher compared to intact eggs. Fertilization in an isotonic NaCl solution resulted in a higher number of developing embryos for albino males (*p* < 0.0005), a non-significant rise for normal-colored males (*p* = 0.002), and a decrease in the mixed group (*p* < 0.0005) compared to fertilization in the water of washed eggs. The use of 100% ovarian fluid as an activation medium resulted in almost 100% fertilization success in all cases: albino, normal males, or mixed. The share of albino embryos in the mixed group reached almost half of the total embryo count (*p* = 0.002). Using higher concentration of spermatozoa (adding of 5 µL sperm to the activation medium (1,500,000 spermatozoa per egg) resulted in high fertilization in all cases with no great difference between treatments (Figure 1b). Nevertheless, the share of albino embryos in the group with fertilization in ovarian fluid was again the highest compared to other treatments and did not significantly differ from the expected “ideal” 50% division (*p* = 0.12).

### 2.2. Ovarian Fluid Prolongs the Motility of Spermatozoa

**Figure A1:**
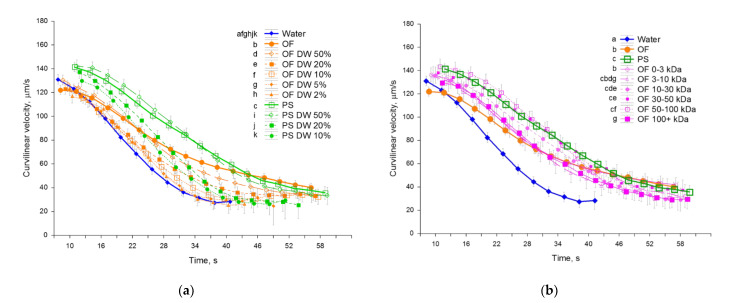
Descriptive statistics data on curvilinear velocity of rainbow trout spermatozoa activated in the presence of ovarian fluid (OF) and other conditions, depending on time post-activation: (**a**) velocity in water, ovarian fluid, NaCl solution isotonic to ovarian fluid (physiological solution, PS, 290 mOsm/L); and their dilutions with water (50%, 20%, 10%, 5%, and 2%); (**b**) motility in MWCO fractions of ovarian fluid. Data are mean values (*n* = 16 for OF and 12 for PS dilutions; *n* = 26 for OF, PS and water; *n* = 11 for MWCO fractions), vertical bars denote 0.95 confidence interval; different letters in the legends denote significant differences assessed by factorial repeated measures ANOVA with correction for multiple comparisons, *p* < 0.001.

Our results confirmed the significant effect of ovarian fluid on the velocity of rainbow trout spermatozoa (Figure A1 in Appendix A) but not for an increase in the initial motility period, which was reported widely (details in analysis by Myers et al. [20]). In these experiments, rainbow trout spermatozoa were fully activated either in a hypotonic medium (water) or an isotonic medium (ovarian fluid or physiological solution). Ovarian fluid improved the longevity of spermatozoa, and the changes in velocity in isotonic saline differed from both the above media. To characterize these differences numerically, we performed a regression analysis of the experimental data.

**Figure 2:**
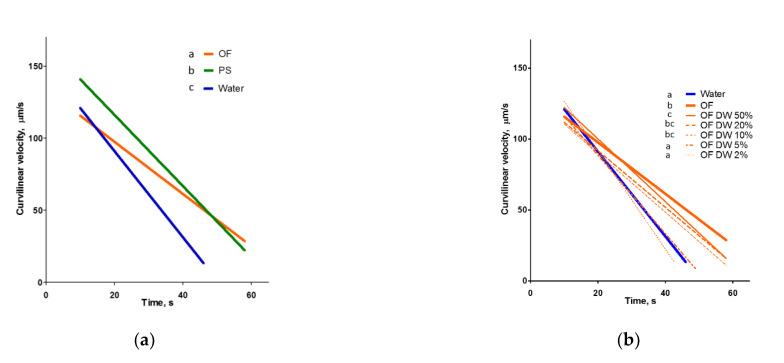

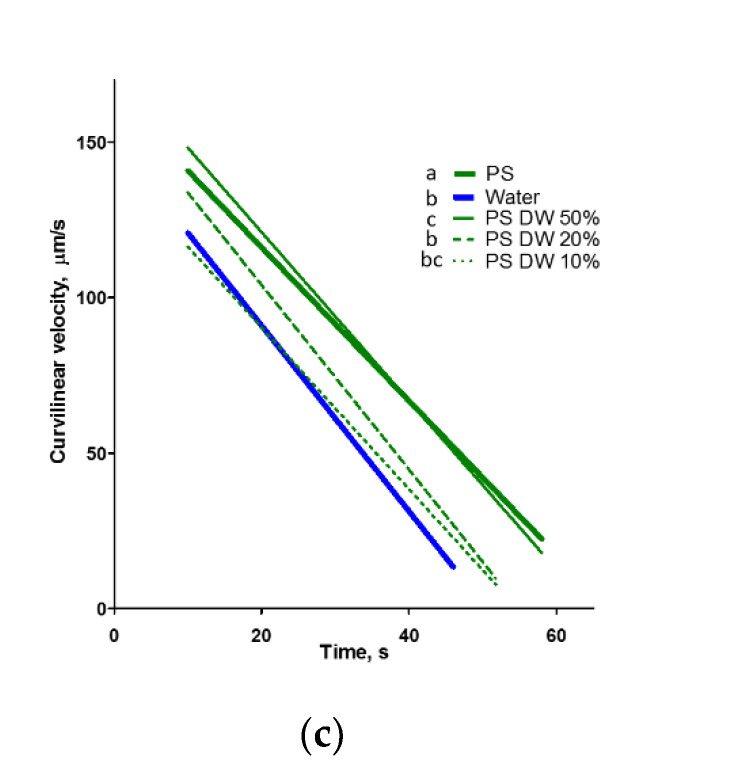
Ovarian fluid prolongs the motility of rainbow trout spermatozoa. The graphs represent changes in the curvilinear velocity of spermatozoa activated in ovarian fluid (OF) and other conditions depending on time post-activation: (**a**) velocity recorded in water, ovarian fluid and NaCl solution isotonic to ovarian fluid (physiological solution, PS, 290 mOsm/L); (**b**) dilutions of ovarian fluid; and (**c**) PS with water. Data are linear regressions of experimental dependencies (shown in the Figure A1 in the Appendix A), the parameters of fitting, values of slopes and intercepts are shown in Table A1. Different superscripts denote significant differences between the slopes of regression lines in one graph, tested with ANCOVA analysis and corrected for multiple comparisons, *p* < 0.001.

The linear regressions of average velocity changes during the motility period in all experimental conditions were characterized by high R^2^ (min 0.8, max 0.95; mean 0.88, SD 0.04, *n* = 17). Thus, these lines may characterize velocity changes during the post-activation time (Figure 2), and axis intercepts may serve as characteristics to compare the initial velocity and longevity in different treatments. The indices characterizing the linear regressions are shown in Table A1 in the Appendix A: the coefficient of determination (R^2^), the slopes, and the intercepts of the x (time) and y (velocity) axes. According to these data, the highest initial velocity was observed in the isotonic NaCl solution, while the lowest was observed in the ovarian fluid. The period of sperm motility was the lowest in water and the highest in ovarian fluid: estimated longevity was almost 47, 67, and 74 s in water, isotonic saline, and the ovarian fluid, respectively (Table S1).

The dilution of ovarian fluid with water led to the disappearance of its longevity enhancing effect: if the content of ovarian fluid was 5%, there was no significant difference (*p* = 0.88) in the regression line slope (Figure 2b, Table A1) compared with that of water. If the spermatozoa were activated in the salines with descending osmolarity, the resulting velocity changes were different from the dilutions of ovarian fluid (Figure 2c, Table A1).

### 2.3. Ovarian Fluid Straightens Trajectories and Has a Trapping Effect on Rainbow Trout Spermatozoa

**Figure 3:**
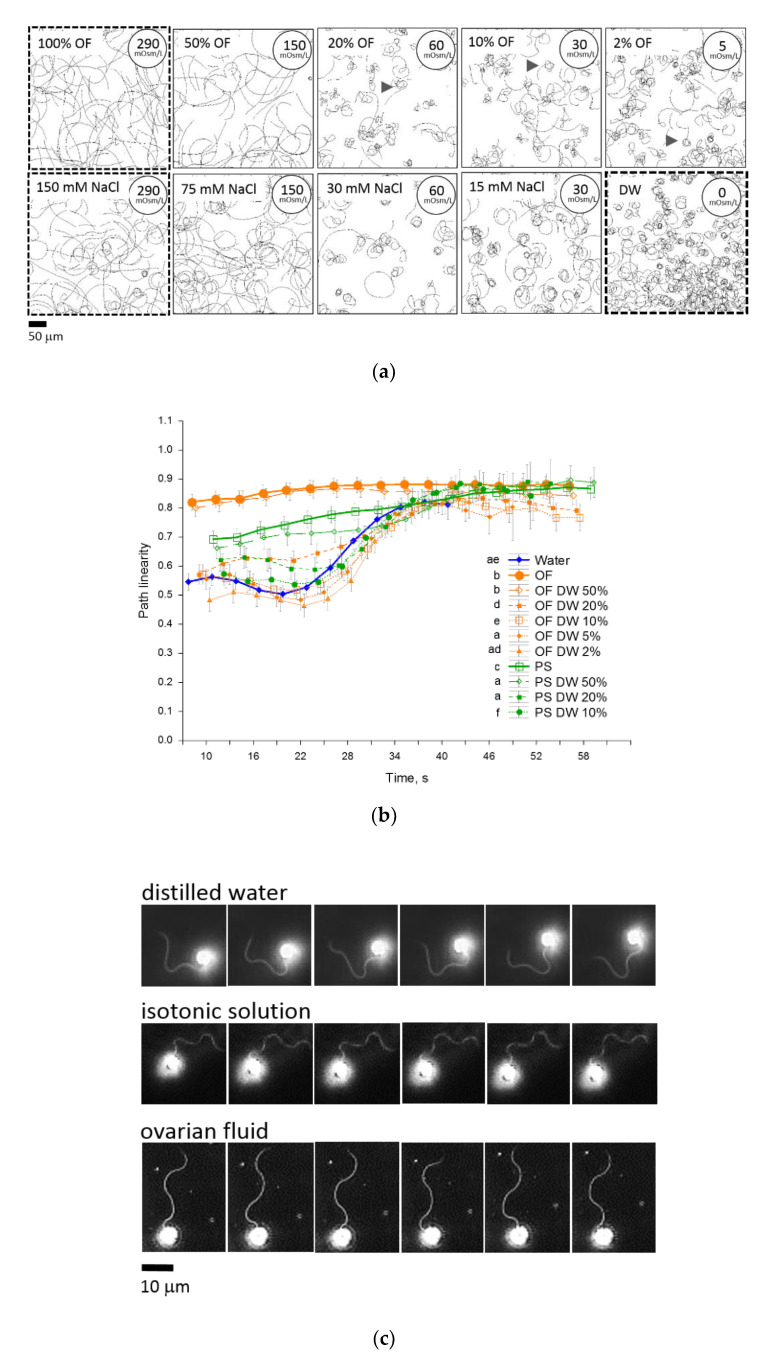

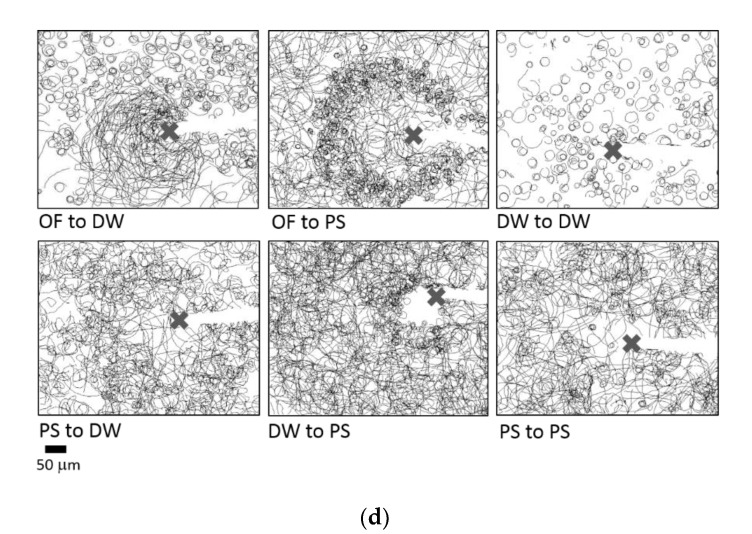
Ovarian fluid straightens trajectories and has a trapping effect on rainbow trout spermatozoa. (**a**) Trajectories visualized from 10 to 12 s post-activation in distilled water (DW), ovarian fluid (OF), NaCl solution isotonic to ovarian fluid (physiological solution, PS, 290 mOsm/L), and their dilutions with water (50%, 20%, 10%, 5%, and 2%). (**b**) The graph shows the linearity of swimming paths of rainbow trout spermatozoa activated in water, ovarian fluid, isotonic saline, and OF dilutions with water (50%, 20%, 10%, 5%, and 2%) depending on time post-activation. Data are mean values (*n* = 16 for OF and 12 for PS dilutions; *n* = 26 for OF, PS and water), vertical bars denote 0.95 confidence interval; different letters in the legend denote significant differences, *p* < 0.001. (**c**) The successive frames represent the flagellar beating of rainbow trout spermatozoa activated in distilled water, 0.9% NaCl solution, or ovarian fluid. The frames are extracted every 0.005 s recorded by high-speed video microscopy (at 2000 fps) using a 20× lens and darkfield microscopy. (**d**) Chemotactic sperm accumulation test: the frames show the swimming tracks of rainbow trout spermatozoa activated in various media near the tip of a microcapillary filled with test fluids: ovarian fluid (OF); distilled water (DW); isotonic NaCl saline (PS, 290 mOsm/L). Each track represents 2 s of motility (15–17 s post activation). The cross shows the tip of the microcapillary.

Spermatozoa activated in water moved in tight circles during the initial motility period (Figure 3a), whereas the trajectories of most cells in ovarian fluid and NaCl isotonic solution were straight or arc-like with a larger proportion of straight moving cells in the ovarian fluid, which is reflected in the higher initial average linearity in Figure 3b. A two-fold dilution of ovarian fluid and NaCl isotonic solution did not essentially change path linearity, while further dilution of both activation media dramatically decreased the proportion of straight-moving spermatozoa. Interestingly, in the diluted ovarian fluid, many cells moved in complex coil-like trajectories (arrowheads in Figure 3a). These had features of the “turn-and-run” pattern [24] associated with “explorative behavior” resulting from the effect of a chemotactic agent. There were no such explorative movement patterns observed in NaCl solutions with similar osmolarities.

The flagellum is the only structure that drives and controls the direction of motility. In a motile rainbow trout spermatozoon, it has a typical wave shape (Figure 3c). There are differences in the distribution of the bends in flagella of spermatozoa activated in water, isotonic saline, and ovarian fluid. In ovarian fluid, flagella have symmetric bends that propagate spermatozoa uniformly. In isotonic saline, flagellar waves have a slight asymmetry, and in water, this asymmetry is maximal. These different patterns accord with various modes of propagation in multiple media: straight in ovarian fluid, straight-to-arc-like motion in isotonic saline, and tumbling in distilled water.

We have tested the behavior of spermatozoa in media with spatially varied substances (gradients) using a spermatozoon accumulation test with a microcapillary.

The typical motility patterns of spermatozoa in our microcapillary tests are presented in Figure 3d and Supplementary Video S1. No changes in the usual tumbling behavior of spermatozoa activated in water were observed when the microcapillary injected the same water. When the capillary was filled with ovarian fluid, the male gametes, which came into contact with the injected “cloud”, changed their behavior to a straight-line pattern. Moreover, the affected cells abruptly changed direction upon reaching the border of the cloud; that is, they were trapped in the ovarian fluid, and a “positive taxis” was observed. Injection of ovarian fluid into the suspension of spermatozoa activated in isotonic NaCl resulted in the appearance of the “tumbling layer” of spermatozoa around the cloud, and the part of the cells that entered the cloud moved straight. Changes in the behavior of spermatozoa and trapping were also observed when isotonic saline was injected into the water as an activation medium. Nevertheless, no differences were seen when the activation medium was changed to the same NaCl isotonic saline, which may suggest the presence of a sort of “osmotaxis” in our experimental conditions. Interestingly, the injection of distilled water into the sperm suspension activated in isotonic saline caused “negative taxis”: the cells avoided entering the hypo-osmotic area, tended to stay in the medium with higher osmolarity, and performed abrupt turns on the borders of the “cloud”.

### 2.4. Rainbow Trout Spermatozoa React to an Abrupt Ca^2+^ Rise in Flagella during Turn-and-Run Behavior

Ca^2+^ takes part in the complex membrane cascade controlling the motility of spermatozoa and, most importantly, its direction [25,26]. It has been shown that the chemotactic response of ascidian spermatozoa (i.e., the appearance of a specific “turn-and-run” pattern, similar to what we observed) is associated with a burst-like rise in Ca^2+^ concentration in the bent segment of the flagellum [27].

**Figure 4:**
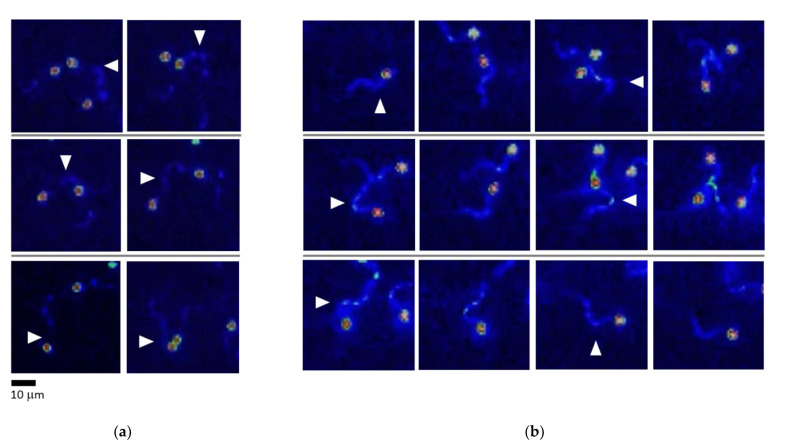
Rainbow trout spermatozoa react to an abrupt Ca^2+^ concentration rise in the flagella during turn-and-run behavior. Fluo-4 fluorescence in the sperm activated in water in the vicinity of the microcapillary filled with ovarian fluid or other fluids. Heat map: blue—minimal Ca^2+^ concentration; red—maximum concentration. (**a**) Frame by frame sequence of cell performing tumbling (shown by arrowheads). (**b**) Frame-by-frame sequence of a sperm cell performing straight run and turns (shown by arrowheads).

A series of experiments similar to the above-described microcapillary tests were performed involving fluorescent microscopy to observe changes in intracellular Ca^2+^ concentration after Fluo4 dye loading and further observation by light excitation at 435 nm. The observation of cells that performed swirling moves in the water did not show any changes in the Fluo-4 fluorescence intensity of their flagella (Figure 4a). In contrast, cells that exhibited a straight run and turn at the boundary of different media (e.g., water and ovarian fluid or water and isotonic saline) showed a bright “burst” of Fluo-4 fluorescence in the bent area of their flagella when turning (Figure 4b), while there were no visible changes in Fluo-4 fluorescence to be seen during a straight run. In other words, there was a local increase in Ca^2+^ concentration during change in motility direction associated with asymmetric flagellar waves.

### 2.5. Isolated Agents of Ovarian Fluid Differently Affect the Behavior of Rainbow Trout Spermatozoa––Effect of Ovarian Fluid Molecular Weight Fractions and Other Protein Solutions

It has been reported that maternal fluids in several species may contain substances that affect spermatozoa, in particular their chemotactic response [28].

What could be the active agent in rainbow trout ovarian fluid in addition to the mentioned above osmotic gradients? To answer this question, we checked the performance of male gametes in several molecular weight cut-off (MWCO) fractions separated from the ovarian fluid, as well as other protein-containing fluids (i.e., thermo-treated ovarian fluid, rainbow trout blood serum, and an aqueous solution of bovine serum albumin).

**Figure 5:**
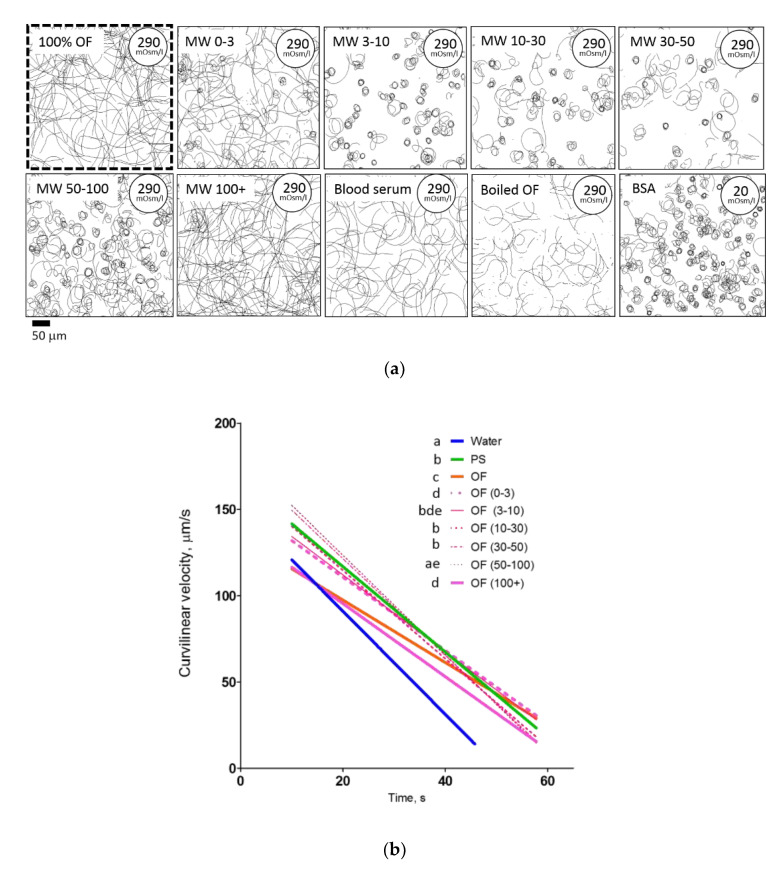

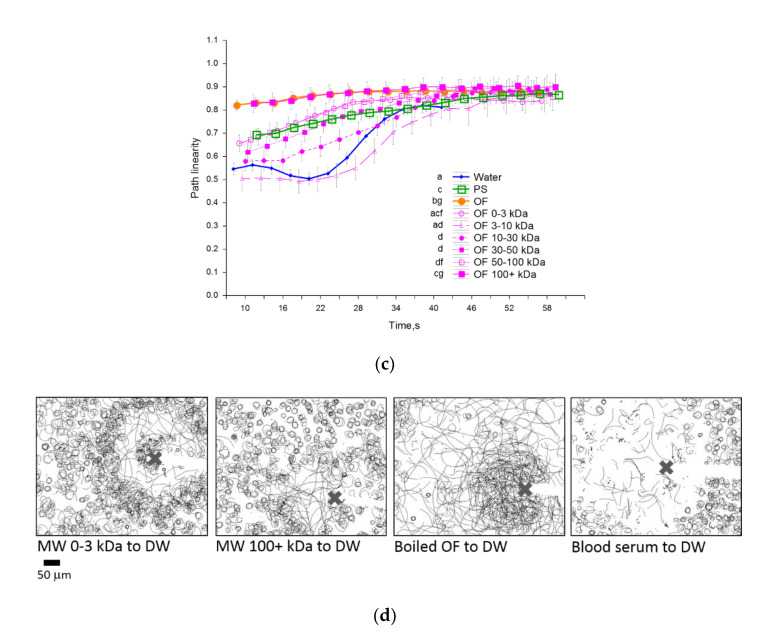
Ovarian fluid molecular weight fractions and other protein solutions affect the behavior of rainbow trout spermatozoa differently. (**a**) Spermatozoa trajectories visualized from 10 to 12 s post-activation in MWCO fractions of ovarian fluid (OF), blood serum, and thermotreated (boiled) ovarian fluid. (**b**,**c**) show the changes in curvilinear velocity and path linearity of the spermatozoa during time post-activation in MWCO fractions of ovarian fluid. Data are VCL linear regressions in (**b**) and path linearity mean values in (**c**); vertical bars denote a 0.95 confidence interval; letters in the legends indicate significant differences, *p* < 0.001. Data are average for 26 males in water, ovarian fluid, and NaCl isotonic saline, and 11 males in MWCO fractions of ovarian fluid. (**d**) Chemotactic sperm accumulation test: the frames show the swimming tracks of rainbow trout spermatozoa activated in distilled water (DW) near the tip of a microcapillary filled with test fluids: MWCO fractions of ovarian fluid with MW 0–3 kDa and 100+ kDa; blood serum and thermotreated ovarian fluid. Each track represents 2 s of motility (15–17 s post activation). The cross shows the tip of the microcapillary.

Among the molecular weight, only the 100+ kDa fraction caused the same motility and path linearity pattern as ovarian fluid (Figure 5a,b). Traits in other MW fractions (0–3, 3–10, 10–30, 30–50, and 50–100 kDa) did not differ significantly from the isotonic NaCl solution. The same motility pattern as in ovarian fluid was found both in rainbow trout blood serum and in thermally treated ovarian fluid. In the aqueous solution of 1 mmol/L bovine serum albumin, the spermatozoa had the same trajectories as in water.

Regarding linear regression slopes, the molecular weight fractions of ovarian fluid were similar to those of the NaCl isosmotic medium, except for the 0–3 and 100+ kDa fractions (Figure 5b).

In the microcapillary cell accumulation test, the low molecular ovarian fluid fraction caused quite a bright response in the behavior of spermatozoa activated in water: some cells swirled in the vicinity of the border of the injected cloud; some spermatozoa moved straight without “escaping” the area, and some cells gathered close to the microcapillary tip (Figure 5c). Other fractions did not differ significantly from the isotonic saline in spermatozoa behavior. Surprisingly, the introduction of blood serum caused inhibition of the motility of cells entering the injected cloud. Introduction of thermo-treated ovarian fluid entailed a bright reaction associated with a significantly visible “belt” (area with less cell concentration, from which the trapped spermatozoa moved closer to the source of the attractant).

### 2.6. Medium Osmolarity and Ca^2+^ Content Have a “Cross Effect” on Motility and Trapping of Rainbow Trout Spermatozoa

**Figure 6:**
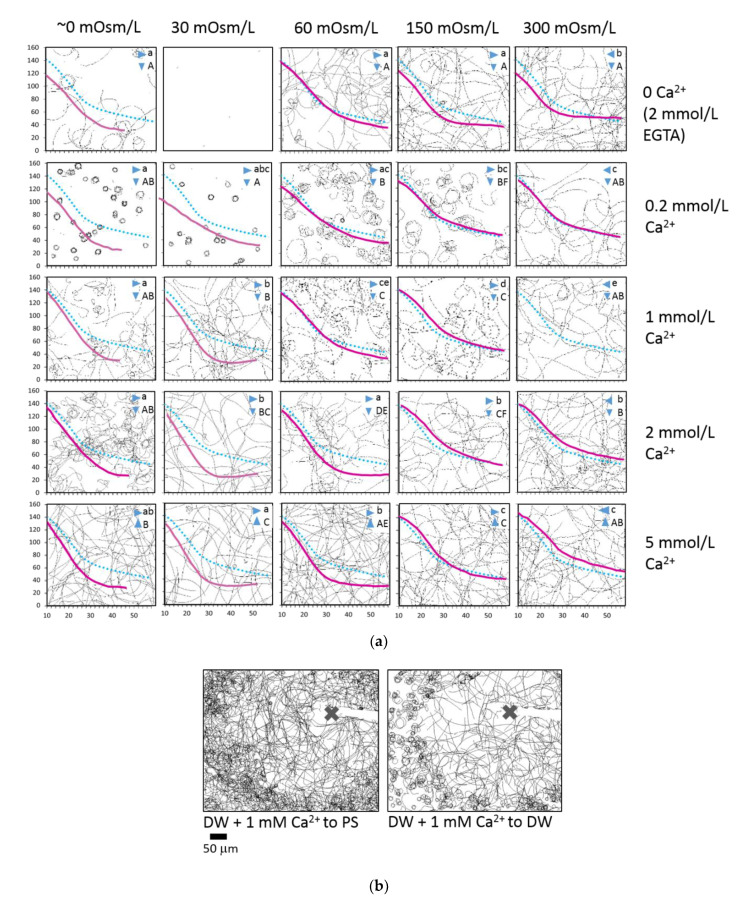
Medium osmolarity and Ca^2+^ content have a cross effect on the motility and trapping of rainbow trout spermatozoa. (**a**) Background: spermatozoa trajectories visualized from 10 to 12 s post-activation in media with osmolarities 30, 60, 150, and 300 mOsm/L, prepared with NaCl and 10 mM Tris buffer, pH 8; and Ca^2+^ content 0, 0.2, 1, 2, and 5 mmol/L prepared with an appropriate amount of CaCl_2_ or 2 mmol/L EGTA in case of 0 mmol/L Ca medium. The “0” mOsm/L medium was distilled water with either 2 mmol/L EGTA or a corresponding CaCl_2_ concentration; i.e., the osmolarity in this medium was, in practice, higher than 0; the actual values are shown in Table 1. Curves: solid lines show the corresponding changes in the curvilinear velocity of the spermatozoa vs. time post-activation. A dotted line is given for reference and shows the recorded velocity in the activation medium with 1 mmol/L Ca^2+^ and 300 mOsm/L osmolarity. Data are mean values (average from eight males). The statistical significance of the differences between VCL dependencies was tested by pair-wise factorial repeated measure ANOVA with a threshold *p*-value of 0.0005. Uppercase letters denote statistical differences in the media with varying Ca^2+^ concentration at the same osmolarity (vertical), and lowercase letters indicate differences for media with varying osmolarity at the same Ca^2+^ concentration (horizontal). (**b**) Chemotactic sperm accumulation test: the frames show the swimming tracks of the spermatozoa activated in distilled water (DW) or isotonic saline (PS) near the tip of a microcapillary filled with distilled water and 1 mM Ca^2+^. Each track represents 2 s of motility (15–17 s post activation). The cross shows the tip of the microcapillary.

The next series of experiments was done to assess the combination of alternating external Ca^2+^ concentration and osmolarity on the motility of rainbow trout spermatozoa and then compare these with the corresponding indices for ovarian fluid. Figure 6a shows the typical tracks recorded at 10–12 s post-activation combined with the corresponding dependences of their curvilinear velocity in media supplemented by Ca^2+^ concentrations, varying from 0 to 5 mmol/L; and osmolarity from 0 to 300 mOsm/L. These indices include the most likely range of osmolarities and Ca^2+^ concentrations met by rainbow trout spermatozoa after release in natural conditions.

It is clear that the motility pattern depends both on the concentration of external Ca^2+^ and the osmolarity of the activation medium. In the absence of external Ca^2+^, the spermatozoa are poorly activated in a 0 mOsm/L medium: they move in circular trajectories. Interestingly, motility in the 30 mOsm/L medium was almost absent. In Ca-free media with 60, 150, and 300 mOsm/L osmolarities, spermatozoa were activated, and most of them had straight trajectories. The curvilinear velocity of spermatozoa was similar in the beginning of the motility period; after 30 s, the cells in media with higher osmolarity had a longer period of motility and higher velocity. The presence of 0.2 mmol/L Ca^2+^ dramatically changed the pattern of motility. The majority of spermatozoa moved in tight circles in media with 0 and 30 mOsm/L osmolarity, and with a rise in osmolarity, the trajectories “uncoiled” and became straighter in the 300 mOsm/L medium. Regarding curvilinear velocity, cells in the media with 60, 150, and 300 mOsm/L osmolarities were faster and motile longer compared to those in the 0 mOsm/L medium. The presence of 1 mmol/L Ca^2+^ in the activation medium straightened trajectories in most cases, even in the 0 mOsm/L medium. A further increase in Ca^2+^ concentration up to 2 and 5 mmol/L did not lead to significant changes compared to the case with 1 mmol/L concentration, except for the medium with 60 mOsm/L osmolarity, where the VCL decreased surprisingly faster compared to media with lower Ca^2+^ content. The velocity graphs show the enhancing effects on velocity and period of motility in media with 60, 150, and 300 mOsm/L osmolarities and 1 mmol/L Ca^2+^, and for 150 and 300 mOsm/L osmolarities supplemented by 2 and 5 mmol/L Ca^2+^.

The presence of Ca^2+^ ions also affects the response of the spermatozoa in the chemotactic microcapillary test, in both hypotonic and isotonic activation media (Figure 6b). In the first case, the cells activated in the water change the motility pattern for a straight one after contact with the injected water containing 1 mmol/L Ca^2+^. The calcium ions in the water injected into the isotonic saline resulted in the absence of “negative osmotaxis” mentioned above: the activated spermatozoa were able to enter the area with the hypotonic medium, and the straight motility did not change.

## 3. Discussion

The present study showed that ovarian fluid produces chemokinetic effects that affect the velocity, linearity, and longevity of rainbow trout spermatozoa. The fluid exhibits a chemotaxis-like or trapping effect on sperm cells, and the male gametes show abrupt changes in the character and direction of motility due to the differences in environmental conditions that tend to follow more optimal conditions. These changes were accompanied by a burst-like rise in the Ca^2+^ content in the curved segment of the flagella. Finally, ovarian fluid had a positive effect on the fertilizing ability of spermatozoa by improving their non-optimal motility. We will discuss how all these fit into gamete encounter guidance and how ovarian fluid changes the outcome of in vitro fertilization.

### 3.1. Rainbow Trout Ovarian Fluid Provides an Optimal Environment for Fertilization

The observation of a positive effect of ovarian fluid on the outcome of in vitro fertilization in salmonids was reported for Caspian brown trout, *S. trutta caspius* [29], and brown trout (*S. trutta f. fario*) [16]. On the other hand, Hugunin et al. [30] recommended washing off the ovarian fluid before artificial fertilization in rainbow trout after finding that it had a negative impact on “sub-fertile females”, which they explained as “preventing fertilization through impeding sperm movement or recognition/contact with the egg”. This adverse reaction might be associated with substances released by overripe or destroyed eggs [31]. Several authors attributed the discrepancies in fertilization outcomes to a post-copulative effect designed to select the best sire: “cryptic female choice”. In particular, Butts et al. [32] found a significant enhancement of spermatozoa performance after ovarian fluid activation in related females. Wojtczak et al. [33] reported that the different effects of ovarian fluid are mainly caused by inter-individual variations in the maternal fluid pH; nevertheless, the authors said that 40% of the variability is caused by other factors, which may include ions, carbohydrates, or proteins. Rosengrave et al. [12] also found that ovarian fluid from various females had different effects on spermatozoa performance in Chinook salmon (*O. tshawytscha*). However, others argue that ovarian fluid in the fertilization medium does not lead to cryptic female choice as in the case of Arctic charr, as reported by Kleppe et al. [34].

Our experiments showed that using ovarian fluid as a fertilization medium significantly enhanced the performance of sperm, which initially showed low motility in water (less than 20% in the albino male samples), with a correspondingly low number of fertilized eggs in the 150,000:1 spermatozoa–egg ratio (Figure 1a). This observation supports the opinions of Rosengrave et al. and Myers et al. [12,20], who emphasized the inadequacy of pre-fertilization motility estimation in salmonids if the test is performed in water.

Interestingly, for non-washed eggs in water as the activation medium, fertilization for unrelated regular-colored males was as low as for related albino males, despite a much higher observed motility percentage for the former in water. Washing off ovarian fluid from albino female eggs significantly enhanced the number of regularly colored embryos (Figure 1a). If ovarian fluid was used as the activation solution, fertilization was maximal just as if isosmotic saline was used to activate the spermatozoa from both types of males. This probably showed that spermatozoa performance may have been triggered by the non-uniformity of the fertilization environment caused by ovarian fluid confining the egg batch. Moreover, using ovarian fluid as a fertilization medium increased the percentage of embryos from related males; that is, the ovarian fluid increased the chances of spermatozoa from the males of the same population winning the sperm competition.

The above findings are valid for one of the two spermatozoa concentrations in our study, i.e., 150,000 spermatozoa per egg in the fertilization medium. Raising this ratio 10-fold to 1,500,000 spermatozoa per egg led to the disappearance of most differences between the experimental cases. This confirmed the importance of adjusting the in vitro fertilization conditions when investigating “tiny” associations and dependencies that may be “masked” by an excess number of male gametes. Moreover, making associations among naturally occurring processes may be inappropriate without exact knowledge of their conditions, e.g., the ratio between gametes.

Thus, the outcomes from in vitro fertilization demonstrated the advantage of ovarian fluid for achieving maximum fertilization. Below, we consider some aspects of sperm behavior that are potentially related to understanding the mechanism of this phenomenon.

### 3.2. Rainbow Trout Ovarian Fluid Enhances the Kinetic Traits of Spermatozoa

Successful fertilization depends on the ability of the male gamete to carry the genetic material and fuse with the female gamete; thus, any change to spermatozoa kinetic characteristics is highly significant. This is especially true of velocity either in the case of a single male without competition from other males [35], or, more particularly, if sperm competition is present: as reported by Levitan [36], the male with faster spermatozoa succeeds against his rivals by passing on a larger relative genetic contribution.

Sperm competition is widespread in externally fertilizing vertebrates, fish in particular [37]. Salmonids are typical species with intensive sperm competitions. Their tactics vary from spawning in pairs in the presence of parasitic or sneaker males to group spawning. The significance of spermatozoa velocity for salmonids has been reported widely (e.g., by Gage et al. and Liljedal et al. [38,39]); in particular, Gage et al. [38] stated that relative sperm velocity was responsible for the competition success of a focal male and that the increased velocity of the “winner’s” spermatozoa allowed a faster discovery of the spawning microenvironment and penetration into the egg micropyle.

Several groups have shown that the presence of ovarian fluid in the activation medium may enhance spermatozoa performance by increasing velocity or longevity [16,19,32,40,41,42]. Interestingly, Butts et al. [32] revealed that the spermatozoa of related (sibling) lake trout (*Salvelinus namaycush*) displayed a rise in curvilinear velocity after activation in 20% water solution of ovarian fluid compared to gametes from an unrelated male. However, Rosengrave et al. [42] did not find any specific individual differences and revealed the absence of correlations between kinetic characteristics in water and ovarian fluid.

In our study, we confirmed that ovarian fluid affected the kinetic characteristics of rainbow trout spermatozoa compared to activation in water. Nevertheless, we did not find any rise in curvilinear velocity 10 s post-activation, which was contrary to the report of Turner and Montgomerie [41] for another salmonid, Arctic charr (*Salvelinus alpinus*), but similar to the case for zebrafish (*Danio rerio*) reported by Poli et al. [19]. As in the latter case, the reduction in rainbow trout spermatozoa velocity post-activation was much lower in the presence of ovarian fluid and its dilution with water compared to plain water: significant differences disappeared only in case of 5% aqueous solution of ovarian fluid (Figure 2b, Table A1). Changes in the curvilinear velocity of spermatozoa activated in isotonic saline differed significantly from those for ovarian fluid. Although the initial velocity of the cells in saline was higher than for water or ovarian fluid, the longevity of spermatozoa activated in the ovarian fluid was higher than for water or isotonic saline. This was in line with the “saving” effect of ovarian fluid reported by Elofsson et al. [43], which found a similar positive effect on sperm longevity in the three-spined stickleback (*Gasterosteus aculeatus*) and associated it solely with the ionic content of the fluid. Interestingly, the dilution of ovarian fluid with isotonic saline led to the absence of significant differences in velocity between diluted (twice or more) ovarian fluid and saline (Figure 2b).

In addition to velocity, the direction of motility tracks plays an essential part in spermatozoa propagation. The ovarian fluid had a prominent effect on the path linearity of the male gametes, which was higher than for water or isotonic saline. A similar observation about a significant rise in path linearity in rainbow trout spermatozoa activated in ovarian fluid was reported by Dietrich et al. [17]. Path straightening in the ovarian fluid was associated with a symmetrical propagation of waves along the flagellum, unlike the non-symmetrical flagellar waves of spermatozoa activated in water (Figure 3c). When decreasing the concentration of ovarian fluid, we observed a rise in specific trajectories, similar to the “turn-and-run” pattern. The latter were frequently observed even in highly diluted ovarian fluid, i.e., 2% (Figure 3a), 5, and 10%, but they were not present in non-diluted ovarian fluid or its 50% dilution. Such behavior was not characteristic of sperm activated in isotonic saline.

Among the molecular weight cut-off fractions of the ovarian fluid, only the 100+ kDa fraction induced a motility pattern that presented no difference with that of the ovarian fluid; i.e., the cells moved almost straight in both cases. In all other MWCO fractions, the trajectories were more circular, and the path linearity presented significant differences with both that of ovarian fluid and the 100+ kDa fraction (Figure 5a,b).

### 3.3. Ca^2+^ Concentration and Osmolarity Have Cross-Effects on Motility Traits

The osmolarity of the medium is one of the essential drivers of freshwater fish spermatozoon motility; in particular, the difference in osmolarity between outer and internal media initializes the function of the membrane orchestra of channels and other molecules, thus triggering the motility [44]. No less critical for motility initiation and its progress are calcium ions, which are an integral part of numerous physiological processes in the spermatozoon [45]. In our experiments on the cross-effects of osmolarity and Ca^2+^ concentration, we varied these indices to identify which among these isolated factors or combination of them may be responsible for the chemokinetic effects of spermatozoa when contacting the ovarian fluid. It is clear that both osmolarity and Ca^2+^ concentration affected path linearity both individually and in combination (e.g., in the absence of external Ca^2+^, the rise in the osmolarity of the activation medium from 0 to 300 mOsm/L entailed the straightening of the spermatozoon trajectories, and a rise in Ca^2+^ concentration from 0 to 5 mmol/L in a hypoosmotic medium caused a similar effect). Remarkably, in the case of an intermediate concentration of Ca^2+^ (0.2 mmol/L), path linearity was highly dependent on the osmolarity of the medium (Figure 6a). Collectively, the combination of Ca^2+^ concentration and osmolarity, corresponding to the ones in the ovarian fluid, controlled motility traits, which was similar to the effect of ovarian fluid; nevertheless, the effect of sole osmolarity was the most significant.

### 3.4. The Ovarian Fluid Causes the Attraction and Trapping of Spermatozoa

The microinjection of fluids into media containing activated spermatozoa is one of the obvious ways to mimic the naturally occurring interface between the water and the ovarian fluid that surrounds the egg. This allowed the behavior of male gametes to be modeled during interaction with maternal fluids, which may differ from the motility characteristics expressed in a uniform environment. In our experiments, rainbow trout spermatozoa were able to respond to the non-uniformity in the medium by becoming trapped within an area that presented more optimal conditions, i.e., osmolarity, pH, and ionic content. In nature, these conditions may be provided only by ovarian fluid; nevertheless, in the experiment, it was possible to investigate how these different factors affected spermatozoa response and which of them is the most critical.

Our experiments showed positive taxis and trapping in the area of plain or diluted ovarian fluid for rainbow trout spermatozoa that had been activated either in water or isosmotic saline. This effect was even stronger toward low molecular weight fractions or thermo-treated ovarian fluid, which was probably caused by an easier spatial distribution of active agents from the injected fluid having a viscosity lower than that in ovarian fluid. It is interesting to note that in some cases, such as that shown in Figure 3d and Figure 4c (and in Supplementary Video S1), when ovarian fluid was injected into an isotonic medium or a low molecular fraction of ovarian fluid was injected into water, spermatozoa separated into several populations, some of which showed a hyperactivation-like motility pattern close to the borders of the injected cloud or near the tip of the microcapillary. Changes in the motility pattern (tumbling to straight run) and trapping were also observed for injections of isosmotic saline or distilled water with 1 mmol/L Ca^2+^ (Figure 3d and Figure 5c); these observations showed that overall, positive taxis toward ovarian fluid results from a complex set of reactions of rainbow trout spermatozoa to various factors in the fluid.

Interestingly, the rainbow trout spermatozoa abruptly changed the direction of their movement (turning-and-running) when they came to the interface between optimal and non-optimal environments. This was exhibited by the trapping behavior after ovarian fluid or isotonic saline was injected into water with activated spermatozoa: the cells tended to stay inside the area, where optimal motility conditions created an extended period of straight motion. Moreover, “negative taxis” behavior was observed when distilled water was injected into the isosmotic activation medium: here, the male gametes “avoided” entering the injected medium (Figure 3d). This behavior allowed us to conclude that rainbow trout spermatozoa were highly sensitive to the environment and could change direction to follow, or to stay in, the optimal conditions, which in nature are most likely provided by ovarian fluid.

### 3.5. Effect of Rainbow Trout Ovarian Fluid on Spermatozoa Is in Line with the Effects of Female Factors in Other Externally Fertilizing Species

The phenomena accompanying the performance of rainbow trout spermatozoa in the presence of female fluids are reminiscent of the previously described triggered motility of marine invertebrates (e.g., sea urchins [46], ascidians [47], or squids [48]). The similarity of these sorts of behavior is even more spectacular considering that the turn-and-run loops in rainbow trout spermatozoa were accompanied by the asymmetric bending of flagella and burst-like rise in Ca^2+^ concentration in the bent flagellar segment (Figure 4). A similar phenomenon was found to mediate the chemotactic activity of ascidian spermatozoa [10], which is associated with the function of membrane chemotactic receptors. As in these marine invertebrates, intraflagellar Ca^2+^ in rainbow trout spermatozoa rises during the change of spermatozoon flagellum symmetry and, respectively, path curvature. If the path curvature is constant, either during a straight run in the ovarian fluid or tumbling in the water, the Ca^2+^ concentration remains constant.

As mentioned above, the vast majority of theories and models of spermatozoon chemotaxis are based on numerous investigations performed in marine invertebrates [9,10]. Among fish, only the sperm–egg encounters of the Pacific herring (*Clupea pallasii*) have been relatively well studied: recently, Yanagimachi et al. [6] presented a prospective model for motility initiation and entry into the egg. The spermatozoa may stay undamaged in the water for several hours, even days, prior to activation, which is a feature unique to herring [49]: the males release their sperm into the water, and the attracted females then release their eggs on the spot. The male gametes are activated in the presence of a specific protein, sperm motility-initiating factor (SMIF), which is associated with the ovarian fluid and egg membrane [50]. The activator molecule interacts with membrane receptors followed by the activation of adenyl cyclase, potassium, and calcium ion channels, and the corresponding accumulation of cAMP and influxes of K^+^ and Ca^2+^ [6]. After being activated, the spermatozoa are attracted to the micropyle area with the aid of another protein, micropylar sperm attractant (MISA) [51]. This attraction is accompanied by the effect of proton pumps and Ca^2+^ channels, which control the intracellular concentration of calcium ions and, correspondingly, the flagella beating pattern [6].

Unlike the Pacific herring, rainbow trout spermatozoa can be activated in water; nevertheless, this activity is extremely short, as it is for most freshwater species. The male (or males) ejaculate at the exact moment, or shortly after the female releases the eggs with plenty of ovarian fluid into the prepared nest. Rainbow trout spermatozoa change their motility pattern in the presence of ovarian fluid: the more that is present in the activation medium, the straighter the path. In the diluted ovarian fluid, the spermatozoa performed explorative “turn-and-runs”, and they had positive taxis to the ovarian fluid. The cells that entered these areas became trapped: if they reached the border with water, they turned back abruptly. This activity is accompanied by the appearance of asymmetric bends in the flagella and a flash-like increase in Ca^2+^ concentration in the bent segment, presumably accompanying the encounter of the spermatozoa with the eggs. These phenomena have certain similarities to the ones described for Pacific herring, as well as for marine invertebrates such as sea urchins and ascidiams, which allowed us to suppose a common basis for chemotactic responses in the broader spectrum of externally fertilizing animals than had been established. The individual peculiarities of the egg (ovarian fluid)–sperm interaction in rainbow trout may be associated with specific features of the spawning process in this species.

## 4. Materials and Methods

### 4.1. Fish Broodstock—Gametes and Fluids Collection

The experiments were performed with gametes of 2–3-year-old rainbow trout (0.6–1 kg) in the spring and autumn of 2017, 2018, and 2019. Altogether, samples from 60 fish were used in this study. Eggs and sperm were obtained during the natural spawning period in the spring or autumn. Fish were obtained from a fish farm in Bušanovice, the Czech Republic, and Oshino Trout Hatchery, Yamanashi Prefectural Fisheries Technology Center, Oshino, Yamanashi, Japan (autumn 2018). The male fish were selected randomly from farm stock, transported in oxygenized water tanks, and kept indoors in a covered water tanks with fresh water and oxygen supply at 11 °C. Sampling was done in all spermiating males once a week. Sperm was collected by stripping, stored on ice during analysis, and used for 6 h. Eggs were collected from batches prepared for artificial propagation at the fish farm during planned fertilization procedures. Ovarian fluid was drained from the eggs with a sieve, centrifuged to remove debris, and stored at 4 °C in plastic tubes or frozen at −80 °C when long-term storage was required. Only ovarian fluid from non-overripe eggs was used in conventional motility analyses. The fertilization test presented in the paper was done in February 2019 using eggs of albino rainbow trout females and sperm from albino and normal-colored males; ovarian fluid from albino females was collected and stored separately.

### 4.2. Preparation of Media

The chemokinetic properties of the ovarian fluid were evaluated in a series of activating media (Table 1): varying concentrations of ovarian fluid (from 100 to 2%); various concentrations of NaCl (Sigma Aldrich, St. Louis, MO, USA) to mimic the osmotic pressure of corresponding dilutions of ovarian fluid; different molecular weight fractions of ovarian fluid; thermo-treated ovarian fluid (boiled at 100 °C for 5 min); rainbow trout blood serum (collected from the blood centrifuged at 3000× *g* for 5 min); and bovine serum albumin (Sigma Aldrich) 1 mg/mL solution in water. The molecular weight cut-off (MWCO) fractions of the ovarian fluid were prepared using Amicon Ultra centrifugal filters with 3, 10, 30, 50, and 100 kDa filters (Merck Millipore Ltd., Carrigtwohill, Ireland). Stepwise centrifugation through the filters was done starting from 100 kDa. Processing was performed at 4 °C until 10 times the concentration of the fluid above the filter (10% of the initial volume) was achieved. The volume of the collected fluids was recovered with an isotonic NaCl (0.9% *w*/*v*) solution. The fluid that was passed through the filter was transferred to the next filter with a lower MWCO, and the procedure was repeated until the lowest MW filter was used.

Additional experiments were performed by checking the cross-effect of Ca^2+^ concentration and osmolarity of the activation media. To do this, a set of media was prepared having varying osmolarity (0, 30, 60, 150, and 300 mOsm/L with NaCl and 10 mM Tris buffer (Sigma Aldrich) at pH 8, where appropriate) and Ca^2+^ (0, 0.2, 1, 2, and 5 mmol/L made with the appropriate amount of CaCl_2_ (Sigma Aldrich) or 2 mmol/L EGTA (Sigma Aldrich)). The “0” mOsm/L medium was distilled water with either 2 mmol/L EGTA or the corresponding CaCl_2_ concentration; i.e., the osmolarity in this medium was, in fact higher than 0. The values are shown in Table 1.

Appropriate solutions were assessed for osmolarity, pH, protein, and ion content. Osmolarity was measured using a freezing point osmometer Osmomat 3000 (Gonotec GmbH, Berlin, Germany) and presented in mOsm/L. Concentrations of sodium and potassium ions were measured by potentiometry using ion-selective electrodes (Bayer HealthCare, Tarrytown, NY, USA). Calcium ion concentration was measured by absorption photometry applying the o-cresolphthalein complexone method [52]. The ion concentration is expressed in mmol/L of the medium. Protein concentration was determined using the Pierce BCA Protein Assay kit (Thermo Scientific, Waltham, MA, USA) and shown in mg/mL. The measurements of the protein and ion content were done in the range of standard calibration curves appropriate to the method used.

### 4.3. Motility Observation and Recording

Sperm suspensions (around 0.1 µL) were carefully mixed for 5 s with 40 µL of tested solutions, and motility (if present) was recorded for 1 min post-activation using the digital camera ISAS (PROISER, Valencia, Spain) or IDS (IDS Imaging Development System GmbH, Obersulm, Germany) set at 25 frames/s and a microscope (UB 200i, PROISER) using a 20× lens and negative phase-contrast. The experiments were performed at 7 °C (supported by a cooling stage (Semic, Krakow, Poland)). Video records were analyzed to estimate spermatozoa motility traits using ImageJ software (U.S. National Institutes of Health, Bethesda, MD, USA) and the following plugins: CASA and CASA modified for multiple analyses [53,54]. The analysis was performed if the decline in the percentage of motile cells did not reach 10%, and only moving cells with a curvilinear velocity higher than 15 αm/s were considered as motile. The values of spermatozoa velocity, linearity of the spermatozoa trajectories, and pattern of motility (1–2 s tracks of individual spermatozoa in the recorded field) were obtained.

### 4.4. Sperm Chemotaxis Tests

A series of experiments were done to assess the response of spermatozoa to the injection of various test fluids (see Table 1) into the activating medium using glass microcapillaries, which represented an accumulation assay conventionally applied for simple spermatozoa chemotaxis analysis [55]. For this purpose, glass microcapillaries (G100, Narishige, Tokyo, Japan) were pooled (PC-100 puller, Narishige) to get microneedles with tips of ≈20 µm openings size, which were additionally cut by a microgrinder (EG-401, Narishige) to obtain uniform tip openings. The microcapillary was filled with the test fluid and assembled with a microinjector (CellTram Vario, Eppendorf, Hamburg, Germany); then, it was fixed on a holder (Narishige) and adjusted above a specimen glass on a microscope stage. The microinjector pressure was applied to ensure a slow fluid discharge. A drop of the activation medium (40 µL) was placed on the glass slide, spermatozoa were activated in the drop by gentle stirring, and the microneedle with discharging fluid was introduced right after the activation of spermatozoa (≈1–2 s). The behavior of the spermatozoa in the vicinity of the tip of the microcapillary was observed directly under the microscope and video-recorded for 1 min. Then, the resulting records were processed by CASA plugin for ImageJ to get the tracks of spermatozoa, and these patterns of motility were analyzed thereafter.

### 4.5. High-Speed Imaging of Spermatozoon Flagellar Beating

The process of a flagellar beating during the motility of spermatozoa in different media was studied using high-speed recording [56]. Motility was recorded during 10–20 s post-activation in water, isotonic NaCl solution, or ovarian fluid using darkfield microscopy (Olympus BX50, Japan). The records were performed by a high-speed video camera (model i-SPEED TR, Olympus, Japan) at 2000 frames per second.

### 4.6. Ca^2+^ Concentration Imaging

To image intracellular calcium ion content ([Ca^2+^]_i_), semen was suspended in 4–5 volumes of immobilization solution, i.e., artificial seminal fluid (Morisawa and Morisawa 1988) containing 0.05% Cremophor EL (Dojindo, Kumamoto, Japan) and 20 μM Fluo-4 AM (Dojindo), and incubated for dye loading at 10 °C for 2 h. Then, the sperm was activated in an observation chamber, and a microinjector with test liquid was introduced. Fluorescence signals emitted by the swimming sperm were captured by a microscope Olympus IX71 (Tokyo, Japan) equipped with fluorescence illumination and a digital CCD camera QImaging Retiga-EXi (Adept Turnkey, Perth, Australia) according to the method described by Shiba et al. [27]. The experiments were done at 12 °C. Then, the videos were analyzed, and the fluorescence of individual cells was tracked using the imaging application TI workbench [57]. The typical fluorescent response observed in spermatozoa during motility was represented in a series of heat maps, where blue denotes the minimum fluorescence of Fluo-4, and red shows the maximum fluorescence.

### 4.7. In Vitro Fertilization

In vitro fertilization assays were performed in February 2019. The fertilization was done with eggs collected from 3 albino females and two mixed-sperm specimens from 5 albino males or 5 normally colored males. The fish were selected randomly, as stated above. The pooling of eggs from various females was designed to minimize the effects of individual male–female interactions [58], which was not an issue in this experiment. The experimental groups are shown in Table 2. The experimental design was designed to determine if the presence of ovarian fluid changed the outcome of in vitro fertilization. In all cases, 5 g of eggs (on average 79 eggs) were fertilized by 0.5 or 5 µL of sperm mixed in 8 mL of water from the hatchery supply system at 11 °C. The concentration of spermatozoa in the sperm was 2.29 × 10^10^/mL: around 150,000 and 1,500,000 spermatozoa per egg, respectively, for 0.5 and 5 µL sperm in the fertilization medium. In some cases, the eggs were washed with 0.9% NaCl solution three times for 10 s to remove the ovarian fluid.

The eggs (washed/non-washed) were put into a plastic beaker, covered with test solution (water, isotonic NaCl solution, or ovarian fluid), and the sperm was added. Then, the beakers were placed on a shaker (around 100 rpm), and after incubation for 1 min, the eggs were rinsed and transferred to glass Petri dishes. Each point was done in three technical replicates.

The dishes were transferred into the tank with baskets for further incubation at 11 °C. The tank had a closed water circuit with aeration, UV treatment, and temperature control. All fertilizations were done in three replicates. The outcome of in vitro fertilization was assessed after 11 days by the number of developing embryos (the development rate was the amount of developing embryos divided by the total number of eggs); simultaneously, the share of albino embryos was counted. The hatched larva was counted as well. The hatching rate (number of hatched larva/number of eggs) did not differ significantly from the number of developing embryos. Thus, only the latter was presented.

### 4.8. Statistical Analysis

The assessment of motility parameters in different activation media was conducted in triplicate for 26 males in water, ovarian fluid, and NaCl isotonic saline (performed as a control during motility and chemotactic tests), 16 males in ovarian fluid dilutions, 12 males in media with varying osmolarity based on sodium chloride, and 11 males in MWCO fractions of ovarian fluid. Sperm samples from 8 males were used for experiments with the combined effects of Ca^2+^ concentrations and osmolarity. Curvilinear velocity (VCL) and path linearity (LIN) were obtained from the motility measurement from 50–300 spermatozoa per replicate per time point during 10–59 s post-activation with 3 s increment. Then, the motility parameters (averaged from technical replicates) were log and logit transformed (VCL and LIN, respectively) to ensure normal data distribution, and an analysis of the interactive effects between variables was performed using repeated measure ANOVA in Statistica v. 13 (TIBCO Software Inc., Palo Alto, CA, USA). Media and post-activation time were considered as within-subjects factors and VCL or LIN were considered as dependent ones. In the case of the calcium ion/osmolarity cross-effect, calcium concentration, medium osmolarity, and time were considered as within-subject factors. Pair-wise analysis was performed to establish significant differences in interaction between media and the time between various activation conditions (i.e., the difference in spermatozoa behavior in multiple media along with motility time). The corresponding correction of *p*-value was done to consider the multiple comparisons; the threshold *p*-value was set to 0.0005.

Then, the data for spermatozoa velocities in several media (water, isotonic NaCl saline, ovarian fluid and dilutions in water, dilutions of isotonic saline with water, MWCO fraction of ovarian fluid) were used to obtain linear regression dependencies in GraphPad Prism version 5 for Windows software (GraphPad Software Inc., La Jolla, CA, USA), and the following parameters were obtained: slope (A), intercepts with x and y axes (B and C), coefficient of determination or the goodness-of-fit (R^2^). The hypothesis for the equality of regression slopes was checked using ANCOVA, and the threshold *p*-value was set to 0.0001.

A binomial model was used to assess significant differences between the groups in the fertilization test. Three technical replicates for each point were pooled together to increase the statistical power of the analysis. The analysis was done using G*Power 3.1.9.6. software [59]. The following hypotheses were tested: (i) if the developing embryo proportions were the same among the groups; (ii) if the proportion of albino embryos was the same between the mixed groups; and (iii) if the proportion of albino and normal color embryos was the same in the mixed groups. Considering the multiple comparisons, the threshold *p*-value was set to 0.0005.

## 5. Conclusions

Thus, in the frame of one investigation, we performed detailed testing of the features accompanying the gamete encounters in a representative of freshwater spawning fish, rainbow trout. These tests revealed the triggering effect of female fluids on the performance of male gametes, which conformed to the features of spawning behavior in these species. We did not find a specific agent responsible for changes to spermatozoa motility; rather, we supposed a combination of several factors. Nevertheless, finding the exact nature of the active agents and the responsive structures in the male gametes is the prospective goal of future studies.

The studying of mechanisms underlying reproductive processes is an important part of evolutionary developmental biology. These mechanisms adapt to the specific conditions of the environment both in the course of evolution and over the lifetime of an individual. These adaptations entail the arising and modification of reproduction strategies and tactics. These are incredibly varied in fish due to the different ecological niches they inhabit. The fact that most fish reproduce externally makes them a good model for performing various experiments in conditions close to in vivo or with a controlled shift of in vivo conditions. This is especially true of freshwater species since, in addition to other factors, their process of fertilization is highly synchronous and limited to several minutes. Thus, the broader inclusion of fish into future studies of gamete encounters will significantly improve the overall understanding of the evolutionary aspects of reproduction, their molecular mechanisms, and their effects on reproductive behavior and vice versa.

## Figures and Tables

**Table 1:** Solutions used for rainbow trout spermatozoa activation and/or chemotaxis tests.

Solution		Used for Activation/Chemotaxis (Injected Fluid) Tests	Osmolarity, mOsm/L	pH
Distilled water		activation/chemotaxis	≈0	-
Tap water		activation/chemotaxis	≈0	-
10 mM Tris HCl buffer		activation	10	8
Ovarian fluid		activation/chemotaxis	290	~8
Ovarian fluid in water	50%	activation/chemotaxis	150	~8
	20%	activation/chemotaxis	60	~8
	10%	activation/chemotaxis	30	~8
	5%	activation/chemotaxis	15	~8
	2%	activation/chemotaxis	5	~8
Ovarian fluid in isotonic NaCl solution	50%	activation/chemotaxis	290	~8
	20%	activation/chemotaxis	290	~8
	10%	activation/chemotaxis	290	~8
	5%	activation/chemotaxis	290	~8
	2%	activation/chemotaxis	290	~8
NaCl solution (+Tris buffer)	150 mmol/L	activation/chemotaxis	300	8
	75 mmol/L	activation/chemotaxis	150	8
	30 mmol/L	activation/chemotaxis	60	8
	15 mmol/L	activation/chemotaxis	30	8
EGTA supplement to water	5 mmol/L	activation/chemotaxis	10	-
Ca^2+^ supplement to water	0.2, 1, 2, 5 mmol/L	activation/chemotaxis	1, 3, 6, 15	-

**Table 2:** Effect of ovarian fluid on fertilization performance in rainbow trout, albino vs. regular-color fish: experimental design.

Washing of Eggs	Sperm Activation Medium	Male Fish Color	Procedure
Intact eggs	Tap water	Albino	5 g of eggs were placed into the plastic beaker and supplemented with 8 mL of water together with 0.5 µL sperm, either from albino or from regular color male. In the case of mixed sperm, the albino and normal color male sperm were mixed together, and 0.5 mL were taken from the mixture.
		Regular color	
		Albino + regular color	
Eggs washed thrice with isotonic saline	Tap water	Albino	
		Regular color	
		Albino + regular color	
Eggs washed thrice with isotonic saline	NaCl 0.9% saline	Albino	
		Regular color	
		Albino + regular color	
Intact eggs	100% ovarian fluid	Albino	
		Regular color	
		Albino + regular color	

## Data Availability

The data on motility parameters presented in this study are openly available in FigShare at https://doi.org/10.5061/dryad.tx95x69z8.

## Supplementary Materials

The following are available online at https://www.mdpi.com/article/10.3390/ijms22179519/s1. Video S1: Response of rainbow trout spermatozoa activated in various media to the introduction of various test fluids using microcapillary: ovarian fluid (OF); distilled water (DW); isotonic NaCl saline (PS, 290 mOsm/L); molecular weight cut-off (MWCO) fractions; thermo-treated (boiled) ovarian fluid; blood serum; distilled water with added 1 mM CaCl_2_. The videos represent periods around 10 s post-activation right after activation of spermatozoa and capillary introduction.

## Appendix A

**Table A1:** Parameters of linear regression lines for curvilinear velocity dependencies (a, b, c correspond to Figure 2; d corresponds to Figure 5b) of rainbow trout spermatozoa activated in different media: distilled water (DW); ovarian fluid (OF); ovarian fluid mixed with distilled water (OF concentration 50%, 20%, 10%, 5%, and 2%); NaCl solutions (PS—physiological solution with osmolarity of 290 mOsm/L, diluted with water in the same ratios as ovarian fluid: non-diluted and 50%, 20%, and 10% of PS in water); and molecular weight fractions of ovarian fluid (with MW 0–3, 3–10, 10–30, 30–50, 50–100, and 100+ kDa). R^2^ is coefficient of determination, *p*-value in the table refers to significance of the slope to be equal to zero. Data A, B, and C are mean ± SE; the different superscripts denote significant differences (*p* < 0.001).

**(a)**					
**Medium**	**R^2^**	***p***	**A ± SE (Slope of Regression Line)**	**B ± SE (Intercept with *y* (VCL) Axis)**	**C (Intercept with *x* (Time) Axis)**
Water	0.86	<0.0001	−3.21 ± 0.08 ^a^	151.5 ± 2.1	47.25
OF	0.88	<0.0001	−1.88 ± 0.03 ^b^	139.2 ± 1.2	73.89
PS	0.94	<0.0001	−2.50 ± 0.03 ^c^	166.7 ± 1.0	66.58
**(b)**					
**Medium**	**R^2^**	***p***	**A ± SE (Slope of Regression Line)**	**B ± SE (Intercept with *y* (VCL) Axis)**	**C (Intercept with *x* (Time) Axis)**
Water	0.86	<0.0001	−3.21 ± 0.08 ^a^	151.5 ± 2.1	47.25
PS	0.94	<0.0001	−2.50 ± 0.03 ^b^	166.7 ± 1.0	66.58
PS DW 50%	0.95	<0.0001	−2.72 ± 0.05 ^c^	173.6 ± 1.7	63.75
PS DW 20%	0.90	<0.0001	−3.20 ± 0.08 ^b^	167.9 ± 2.5	52.41
PS DW 10%	0.86	<0.0001	−2.99 ± 0.10 ^bc^	151.5 ± 3.0	50.77
**(c)**					
**Medium**	**R^2^**	***p***	**A ± SE (Slope of Regression Line)**	**B ± SE (Intercept with *y* (VCL) Axis)**	**C (Intercept with *x* (Time) Axis)**
Water	0.86	<0.0001	−3.21 ± 0.08 ^a^	151.5 ± 2.1	47.25
OF	0.88	<0.0001	−1.88 ± 0.03 ^b^	139.2 ± 1.2	73.89
OF DW 50%	0.89	<0.0001	−2.19 ± 0.05 ^c^	144.4 ± 1.8	65.95
OF DW 20%	0.85	<0.0001	−2.01 ± 0.06 ^bc^	130.4 ± 2.2	64.76
OF DW 10%	0.80	<0.0001	−2.16 ± 0.08 ^bc^	130.0 ± 2.9	60.17
OF DW 5%	0.89	<0.0001	−3.03 ± 0.10 ^a^	147.2 ± 2.8	48.51
OF DW 2%	0.87	<0.0001	−3.38 ± 0.14 ^a^	152.5 ± 3.8	45.10
**(d)**					
**Medium**	**R^2^**	***p***	**A ± SE (Slope of Regression Line)**	**B ± SE (Intercept with *y* (VCL) Axis)**	**C (Intercept with *x* (Time) Axis)**
Water	0.86	<0.0001	−3.21 ± 0.08 ^a^	151.5 ± 2.1	47.25
OF	0.88	<0.0001	−1.88 ± 0.03 ^b^	139.2 ± 1.2	73.89
PS	0.94	<0.0001	−2.50 ± 0.03 ^c^	166.7 ± 1.0	66.58
OF (0–3)	0.85	<0.0001	−2.11 ± 0.06 ^d^	152.0 ± 2.3	71.88
OF (3–10)	0.84	<0.0001	−2.36 ± 0.10 ^bde^	157.6 ± 3.5	66.91
OF (10–30)	0.93	<0.0001	−2.66 ± 0.06 ^b^	169.4 ± 2.1	63.59
OF (30–50)	0.93	<0.0001	−2.63 ± 0.06 ^b^	172.1 ± 2.0	65.45
OF (50–100)	0.93	<0.0001	−2.93 ± 0.06 ^ac^	181.6 ± 2.1	62.03
OF (100+)	0.85	<0.0001	−2.21 ± 0.07 ^d^	140.3 ± 2.6	63.43
